# Impulse control disorders in Parkinson disease and RBD: A longitudinal study of severity

**DOI:** 10.1212/WNL.0000000000007942

**Published:** 2019-08-13

**Authors:** Fahd Baig, Mark J. Kelly, Michael A. Lawton, Claudio Ruffmann, Michal Rolinski, Johannes C. Klein, Thomas Barber, Christine Lo, Yoav Ben-Shlomo, David Okai, Michele T. Hu

**Affiliations:** From the Oxford Parkinson's Disease Centre (F.B., M.J.K., M.A.L., C.R., M.R., J.C.K., T.B., C.L., Y.B.-S., D.O., M.T.H.), and Nuffield Department of Clinical Neurosciences (F.B., M.J.K.), University of Oxford; Population Health Sciences (M.A.L., Y.B.-S.) and Translational Health Sciences (M.R.), University of Bristol; and Department of Psychological Medicine (D.O.), Oxford University Hospitals NHS Trust, UK.

## Abstract

**Objective:**

To describe the prevalence, natural history, and risk factors for impulse control behaviors (ICBs) among people with Parkinson disease (PD), those with REM sleep behavior disorder (RBD), and controls.

**Methods:**

Participants with early PD (within 3.5 years of diagnosis), those with RBD, and controls were clinically phenotyped and screened for ICBs longitudinally (with the Questionnaire for Impulsivity in Parkinson's Disease). ICB-positive individuals were invited for a semistructured interview, repeated 1 year later. The severity of the ICB was assessed with the Parkinson's Impulse Control Scale. Multiple imputation and regression models were used to estimate ICB prevalence and associations.

**Results:**

Data from 921 cases of PD at baseline, 768 cases at 18 months, and 531 cases at 36 months were included, with 21% to 25% screening positive for ICBs at each visit. Interviews of ICB screen–positive individuals revealed that 10% met formal criteria for impulse control disorders (ICD), while 33% had subsyndromal ICD (ICB symptoms without reaching the formal diagnostic criteria for ICD). When these data were combined through the use of multiple imputation, the prevalence of PD-ICB was estimated at 19.1% (95% confidence interval 10.1–28.2). On follow-up, 24% of cases of subsyndromal ICD had developed full symptoms of an ICD. PD-ICD was associated with dopamine agonist use, motor complications, and apathy but not PD-RBD. ICD prevalence in the RBD group (1%) was similar to that in controls (0.7%).

**Conclusions:**

ICBs occur in 19.1% of patients with early PD, many persisting or worsening over time. RBD is not associated with increased ICD risk. Psychosocial drivers, including mood and support networks, affect severity.


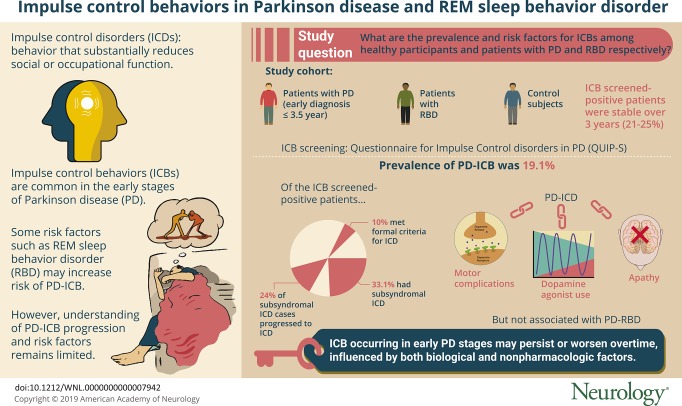


Impulse control behaviors (ICBs) in Parkinson disease (PD) are a range of behaviors linked by their reward-based, repetitive natures (hereafter referred to collectively as ICBs including the related behaviors). Impulse control disorders (ICDs) are defined as behaviors with sufficient impact on social or occupational function to meet “caseness” for disorder, while subsyndromal ICDs comprise the remainder of the full spectrum of ICBs. Most studies have taken a categorical rather than dimensional approach to the presence of ICBs despite the observation that these behaviors are common, dimensional in nature, and variably associated with distress. To date, this has limited our understanding of the risk factors for and natural history of ICBs. For instance, it is still unclear whether the subsyndromal ICDs convert to full ICDs, remit, or continue in their subsyndromal forms.

Prevalence estimates of PD-ICBs vary between 14% and 40%,^1–6^ with part of the variability due to the absence of gold-standard semistructured interviews that conform to DSM-5 (or DSM-5 aligned) diagnostic criteria. In addition, a lack of prospective studies limits our understanding of the course and prognosis of PD-ICBs.

Certain risk factors have been identified, including dopaminergic medication, predominantly dopamine agonists (DAs), along with demographic and disease-specific factors such as the presence of motor complications. In addition, while the presence of REM sleep behavior disorder (RBD) may infer a higher risk of developing PD-ICD,^7,8^ it is not known whether RBD itself or whether a particular RBD-PD subtype confers an increase risk.^9^

We aimed to address the following questions: What is the distribution and severity of PD-ICBs, and how does this vary over time? What is the expected prevalence of PD-ICBs? Finally, which clinical factors are associated with PD-ICBs?

## Methods

### Participants

This study was nested within the Oxford Parkinson's Disease Centre Discovery cohort, a large, multicenter, UK-based prospective study of patients with early PD under longitudinal follow-up. Full details of the protocol have been described elsewhere.^9–11^ In brief, patients with PD diagnosed within 3.5 years were recruited to the cohort between September 2010 and September 2014. Cases were eligible for inclusion if they met the UK PD Brain Bank criteria for diagnosis^12^ regardless of their age at onset, family history, or cognitive status. Follow-up visits were every 18 months, at which point the diagnosis was reviewed by a specialist in movement disorders.

The control population, recruited from spouses and general public volunteers in the same region, were assessed to ensure that they did not have PD or a first-degree relative with PD. The RBD group comprised participants with a diagnosis made by clinical assessment and polysomnography according to standard International Classification of Sleep Disorders-II criteria.^13^ Participants were excluded from this group if their symptoms were judged to be due to medications or associated with another neurologic condition.

At each visit, clinical assessments were performed with validated tools to assess a broad range of motor and nonmotor symptoms. These assessments included cognition (Montreal Cognitive Assessment); personality (Big Five Inventory); orthostatic hypotension (lying and then 3-minute standing blood pressure); hyposmia (the 16-stick Sniffin’ Sticks odor identification test); RBD (Rapid Eye Movement Sleep Behavior Disorder Screening Questionnaire); dexterity (Purdue Pegboard Test); and the Movement Disorder Society–revised Unified Parkinson's Disease Rating Scale (MDS-UPDRS). During the study period, the more commonly used Hospital Anxiety and Depression Scale replaced the Leeds Anxiety and Depression Scale for mood assessment. Hospital Anxiety and Depression Scale scores were calculated with the Leeds Anxiety and Depression Scale when missing with standard equipercentile methods, which we have published previously, for consistency and ease of interpretation.^14,15^

### Assessment of ICBs

At baseline and then every 18 months, all participants completed the Questionnaire for Impulse Control Disorders in Parkinson's Disease (QUIP) Short Form (QUIP-S^16^) to identify participants with possible PD-ICD. All participants screening positive on their most recent QUIP assessment were invited to an interview between September 2015 and June 2016. Each participant was invited to participate in a semistructured interview either by telephone or in clinic, depending on patient preference.

The Parkinson's Impulse-Control Scale (PICS^17^) is a clinician-rated scale based on a semistructured interview used to measure both the frequency and impact of a range of ICBs and to provide an index of severity. Seven ICBs are included in the PICS; gambling, shopping, eating, hypersexuality, simple (punding) and complex (hobbyism) repetitive behaviors, and compulsive overuse of medication (dopamine dysregulation syndrome), with or without off-period dysphoria. It has been validated in PD, has high interrater reliability, and has been proven sensitive to change.^17^ For each ICB, structured questions specific to the behavior are used to collect information on the intensity of the behavior (frequency and scale of behavior, score 1–4) and its riskiness and impact on the individual and others (e.g., financial or social effects, score 1–3). Higher scores denote greater severity. Intensity and impact scores are multiplied to give a single severity score with a total severity indicated by the sum of the 7 ICBs.

Two clinicians with an interest in movement disorders performed the interviews after training from a neuropsychiatrist with a special interest in these disorders (D.O.), recording both quantitative and qualitative information. Interviewers discussed all cases potentially symptomatic with an ICB with both the other interviewer and the neuropsychiatrist to confirm the diagnosis. To meet criteria for a PD-ICB, behaviors needed to be de novo in nature or judged as an exacerbation of preexisting behaviors after administration of dopaminergic medication. We use the term subsyndromal ICD to describe cases with symptoms of ICB that were not severe enough to meet the diagnostic criteria for PD-ICD. ICD (in cases without PD) or PD-ICD was diagnosed with DSM-5^18^ or published criteria for PD-ICD.^19–22^

If a patient was found to have PD-ICD, then the patient’s physician, specialist nurse, and family practice doctor were informed with the patient's permission. In addition, an age- and sex-matched sample of participants who screened negative for ICB were also invited for the more in-depth interview so that we could ascertain the sensitivity and negative predictive value of the screening methodology.

All participants found to have ICB after the in-depth interview were invited to an identical follow-up interview 12 to 18 months later.

### Statistical analysis

We estimated the prevalence of PD-ICB in 2 ways. First, we assumed that the prevalence of PD-ICBs was the same in the participants who participated in the in-depth questionnaire and those who were invited but were not interviewed (nonresponders). Then we used multiple imputation with 25 imputed datasets to account for the missing data on caseness for the nonresponders. This allowed us to examine for any potential bias due to nonresponse to the interview invitation.

We then used logistic regression to determine which clinical variables were associated with PD-ICBs. Variables with crude associations giving a value of *p* < 0.2 were carried over to calculate adjusted associations in a multivariable model. We then used backward stepwise selection to include all variables associated with values of *p* < 0.05 in the final model. The clinical data collected at the time of interview and measurements from the corresponding study visit were used in the analysis of the in-depth interview.

### Data availability

Applications for deidentified data can be made to the Oxford Parkinson's Disease Centre (opdc.ox.ac.uk/external-collaborations).

### Ethics approval and consent

We obtained informed consent from each participant. Ethics approval for this study was granted by the Berkshire Ethics Committee, South Central, National Research Ethics Service (United Kingdom, reference No. 10/H0505/71).

## Results

**Figure 1:**
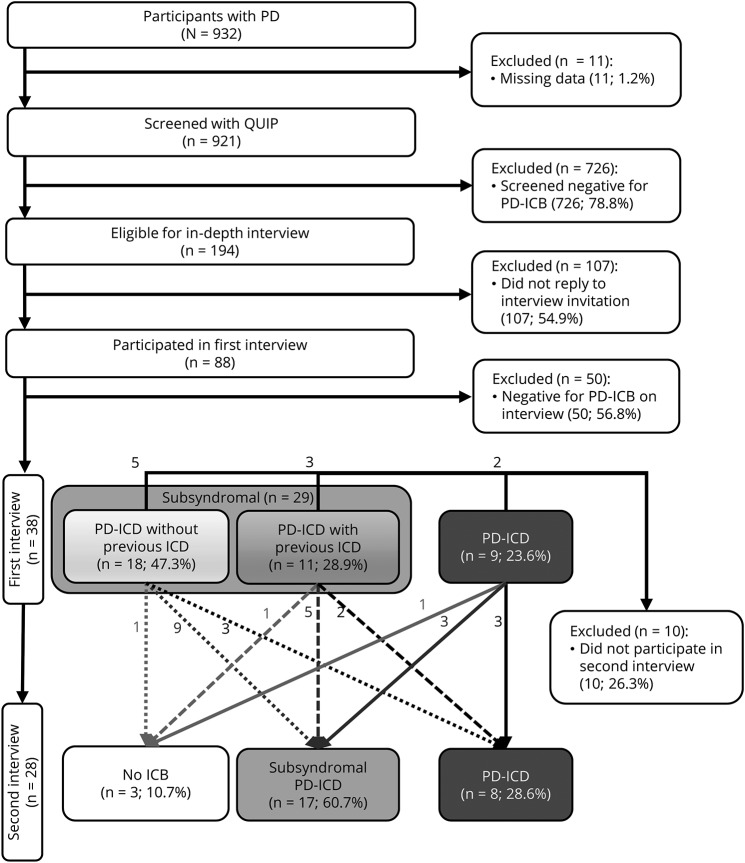
Participant selection and results from in-depth interviews This figure illustrates the selection process for in-depth interviews with the Questionnaire for Impulse Control Disorders in Parkinson's Disease (QUIP) screening tool and demonstrates the changes in impulse control behavior (ICB) category between the first and second interviews. Participants with subsyndromal impulse control disorder (ICD) at the first interview are separated into 2 categories: those who had previously met the criteria for PD-ICD (retrospectively) and those whose symptoms had always been subsyndromal ICD. PD = Parkinson disease.

By January 2018, 932 cases of PD had been recruited; 11 (1.2%) had missing data and were excluded. Follow-up data were available for 768 of 932 (82%) cases at 18 months and 531 of 932 (57%) at 36 months. Attrition was due to a combination of withdrawal from the study, death, or participants still awaiting follow-up. Study participants who had screened positive for ICD at their most recent visit on September 29, 2015, were invited to the in-depth interview. Figure 1 provides a flowchart of the selection process, and table 1 gives the demographics of included participants. Full baseline features have been published previously.^9^

**Table 1:**
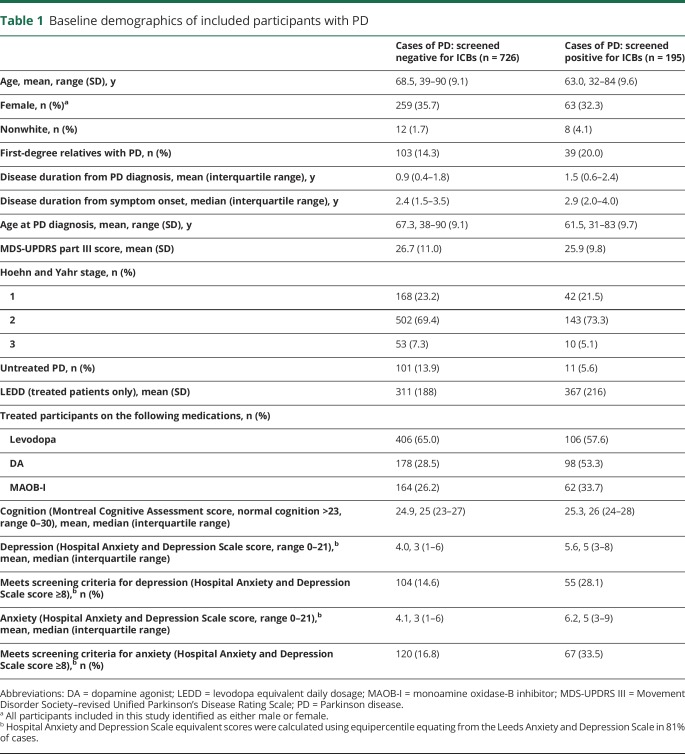
Baseline demographics of included participants with PD

### Distribution of ICBs over time with a screening tool

According to the QUIP-S screening tool, 21% (195 of 921, 95% confidence interval [CI] 18.6–24.0) of the PD group reported symptoms of ICB at baseline. On follow-up, 24% (184 of 768, 95% CI 21.0–27.1) screened positive at 18 months, and 25% (133 of 531, 95% CI 21.4–29.0) screened positive at 36 months. The number of participants with PD reporting >1 PD-ICB also remained stable over the follow-up visits. At baseline, this was 8.3% (76 of 921, 95% CI 6.6–10.2); at 18 months, 8.3% (64 of 768, 95% CI 6.5–10.5); and 36 months, 9.6% (51 of 531, 95% CI 7.2–12.4).

**Figure 2:**
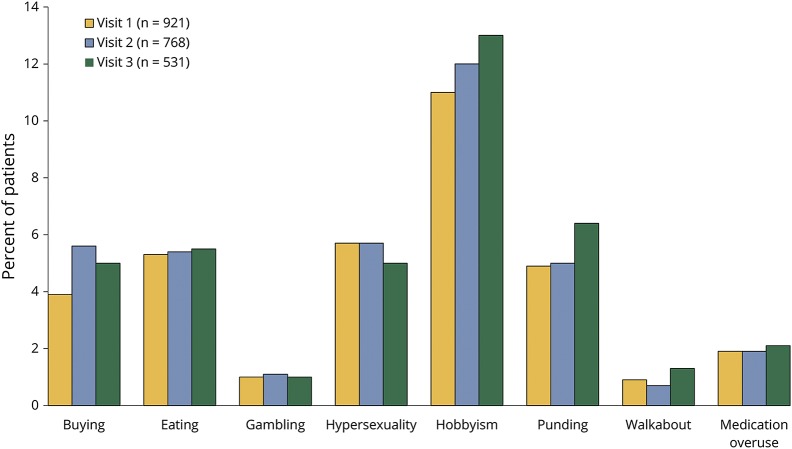
Categories of ICB found on screening at baseline and follow-up This figure demonstrates the percentage of cases of Parkinson disease (PD) screening positive for each impulse control behavior (ICB) at each of the first 3 visits, each ≈18 months apart. If an individual had screened positive for more >1 PD-ICB, each positive result has been included.

The distribution of PD-ICB domains was largely unchanged over the 3-year follow-up period (figure 2). Hobbyism was the most common PD-ICB domain, with gambling and walkabout the least common. Compulsive eating, shopping, and hypersexuality were reported at similar frequencies.

### Distribution and severity of ICB with an in-depth interview

There were no significant differences in the demographics of the cases who responded and those who did not respond to the invitation to the in-depth interview. Table 2 summarizes the results of the in-depth interview, with 10% (9 of 88) meeting the criteria for PD-ICD and 33% (29 of 88) consistent with subsyndromal ICD.

**Table 2:**
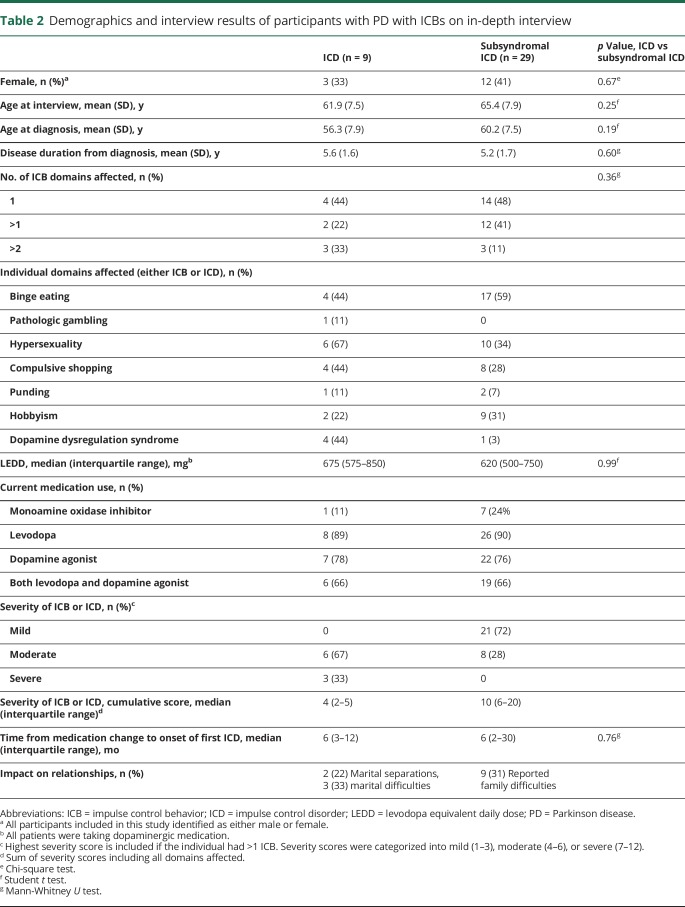
Demographics and interview results of participants with PD with ICBs on in-depth interview

Two participants had symptoms of ICB that predated their diagnosis of PD and had remained unchanged during adulthood; thus, they were not considered to have PD-ICB. The remaining 48 participants did not have symptoms of PD-ICB. To check the negative predictive value of the screening method, an age- and sex-matched sample of 89 participants with PD who screened negative on the QUIP were invited to interview, of whom 48% (43 of 89) took part. None of those interviewed had PD-ICD, but 3 had symptoms of subsyndromal PD-ICD (negative predictive value 93%).

A similar proportion of the control group (21%, 61 of 295) and a slightly higher proportion of the participants with RBD (34%, 35 of 102) screened positive for ICB. Of these, 52% (32 of 61) of the control group and 49% (17 of 35) of the RBD group participated in the in-depth interview. In the control group, 2 (0.7% of total) participants met the criteria for a compulsive eating disorder, one of whom also had a diagnosis of attention-deficit/hyperactivity disorder. In the RBD group, 1 participant (1% of total) met the criteria for binge eating disorder, which had been long-standing without any noticeable association of severity since the diagnosis. No other ICDs were determined.

Binge eating disorder was the most common domain in the in-depth interview, followed by hypersexuality, hobbyism, and compulsive shopping. While the QUIP seemed to overestimate hobbyism, the domains were otherwise similar in proportion.

Subsyndromal PD-ICDs were more common than ICDs. There were no clear differences in the characteristics of the 2 groups. More than half the cases with either subsyndromal ICD or ICD had symptoms in >1 domain. The total levodopa equivalent dailydose (LEDD) and types of dopaminergic medication used were broadly similar in each group. While binge eating was the most common finding in subysndromal ICD (59%, 95% CI 39–76), hypersexuality was the most common PD-ICD (66%, 95% CI 30–93). However, the small absolute numbers make comparisons between groups difficult.

Severity scores were higher in the PD-ICD group, as would be expected. This reflects not only the increased severity of each individual domain but also the additive nature of multiple domains involved in PD-ICD.

However, it is noteworthy that 28% (8 of 29) of cases with subsyndromal ICD had a moderate severity score in their worst affected domain, indicating a notable effect on their well-being in excess of what would be expected in the subsyndromal category. Each of these cases had symptoms that had until recently been consistent with PD-ICD but, at the time of interview, had improved to the extent that they no longer fulfilled the full criteria. Despite the improvement in the intensity of the symptoms, the impact of the more severe symptoms was ongoing (e.g., weight gain due to compulsive eating and the social ramifications of hypersexuality). The detrimental effects of subsyndromal ICD in itself, though, were important to the individual, with almost 1 in 3 cases reporting family difficulties as a result of this behavior.

Symptoms of PD-ICD tended to occur relatively soon after the patients started dopaminergic medication, while there was a greater range in time to symptom onset in the subsyndromal ICD group, although this did not meet the threshold for significance on formal testing.

We recorded a summary of each interview to allow post hoc qualitative data analysis, which revealed some recurring themes. An observed trend was that at least 13 in the subsyndromal ICD group described that their change in behavior was consistent with an exaggeration of long-standing behaviors. The most common example was binge eating disorder; 10 of 17 cases described that they had a tendency to binge eat before being diagnosed with PD, often in association with stress. This contrasted with the participants with full ICD, of whom only 2 described similar symptoms before PD onset and would describe their symptoms as new or out of character.

### Follow-up in-depth interviews of cases of PD with ICB

At follow-up, 74% (28 of 38) of the cases of PD with ICB were interviewed again after a minimum of 12 months (median follow-up 15.6 months). The changes in ICB category between the first and second interviews are illustrated in figure 1. Cases maintained their ICD or subsyndromal ICD status, improved to asymptomatic or subsyndromal ICD, or progressed to PD-ICD from subsyndromal status. Those in the last category who had been in remission are considered to have relapsed into PD-ICD.

**Figure 3:**
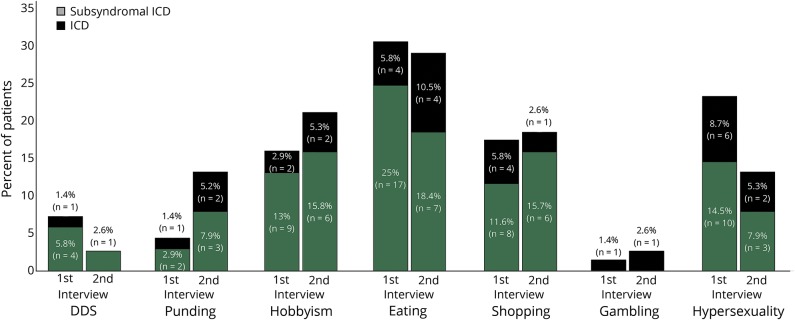
Categories of ICB at baseline and follow-up interview Percentage frequency of impulse control behavior (ICB) domains in the in-depth first interview (n = 38) and follow-up interview (n = 28) (several participants exhibited >1 ICB). DDS = dopamine dysregulation syndrome; ICD = impulse control disorder.

Proportionally, symptoms of hypersexuality and dopamine dysregulation syndrome seemed to improve between the 2 interviews, while punding seemed to become more common (figure 3). Fourteen participants (50%) experienced an improvement in the severity score of their most severe ICB; 6 (21%) remained stable; and 8 (29%) experienced a worsening in this score.

Only 1 participant (of the 23 taking DAs) had withdrawn the DA completely in the interval between first and second interviews. No participants were newly started on DA. Four cases had stopped their DA recently before the first interview. Of the 5 subsyndromal ICD cases who had ceased taking a DA, 4 experienced a reduction in symptom severity between the first and second interviews, while 1 case experienced an increase.

The average LEDD at the first interview among participants with ICB was 666 (SD 344) mg and at the second interview was 826 (SD 317) mg. The mean LEDD of DAs only in participants with subsyndromal ICD and full ICD was 280 (SD 128) mg at the first interview and 331 (SD 212) mg at the second interview. Changes in LEDD were associated with both increases and decreases in ICB severity. There was no significant relationship between change in LEDD or DA-LEDD and change in ICB severity.

Qualitative data revealed that external factors were reported by participants to directly influence the severity of ICB symptoms. For example, 1 participant described recovering from a prolonged period of low mood (also after a bereavement), and this recovery was accompanied by improvement in her overspending and hypersexual behaviors. Another reported social isolation at the first interview but thereafter had joined several new community groups and expanded her social circle, leading (in her opinion) to resolution of her overspending behavior.

Severity scores could also vary because of changes in impact of the symptoms, while the intensity of the behavior remained the same. Similarly, the impact of a behavior that remained stable in terms of intensity could become more troublesome over time. An example is an increase in appetite, which led to weight gain over time. Conversely, some participants reported that while the urges for their behaviors may still have been present, they and their families had developed a range of coping strategies (including measures like financial control) so that they minimized the effects of the impact.

### Prevalence of PD-ICB and associated symptoms

The estimated prevalence of PD-ICB (including the full spectrum of severity), assuming the same proportion in those who did not have an in-depth interview and those who did, was 14.5% (95% CI 12.4–16.9). The use of multiple imputation gave a slightly higher estimate of 19.1% (95% CI 10.1–28.2), suggesting that nonresponders were slightly more likely to have ICBs.

The association of clinical symptoms with PD-ICB is shown in table 3. DA use, severity of motor complications (MDS-UPDRS IV score), and apathy were associated with PD-ICB in the final model. The area under the curve for the final model was 0.75 (95% CI 0.65–0.84).

**Table 3:**
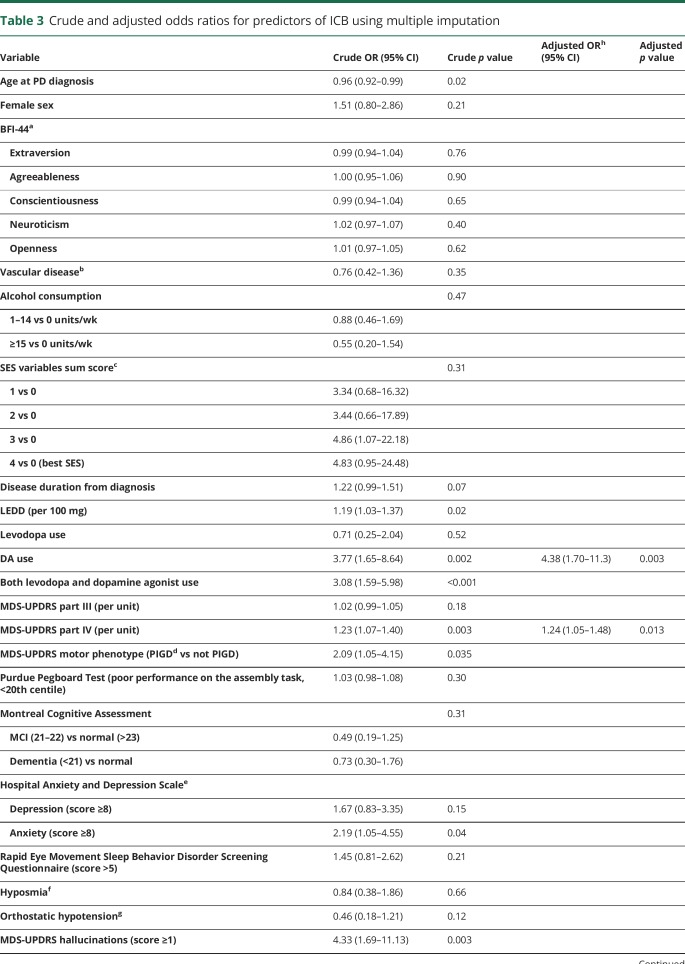

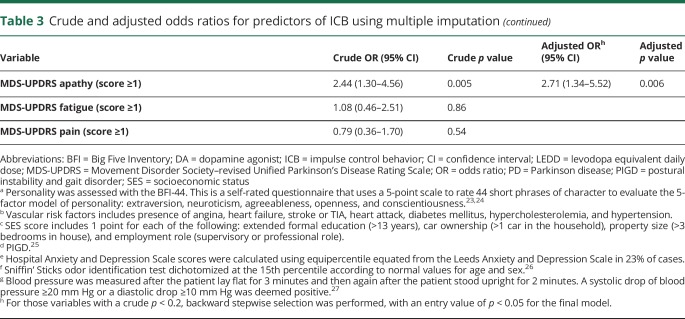
Crude and adjusted odds ratios for predictors of ICB using multiple imputation

## Discussion

This is the first study to perform an in-depth semistructured interview assessing the full range of ICB severity in a dimensional and longitudinal manner. It therefore provides a more granular, clinically relevant understanding of the course and progression of symptoms compared with previous categorical approaches to ICD presence. ICBs are common in the early stages of PD (19.1% prevalence), with a larger proportion of this population having symptoms of subsyndromal ICD without meeting the established criteria for a disorder. In addition to DA use, motor fluctuations and apathy are clinical predictors of ICB. RBD, in the absence of PD or another neurodegenerative disorder, is not in itself associated with a greater risk of ICD. Despite notification of the patient and the care teams, most of the participants with these symptoms continued to be symptomatic a year later, with a proportion of those with subsyndromal ICD going on to develop the more severe disorder.

### Prevalence of ICBs

The results of the QUIP in this cohort (21%–25% screening positive across 3 years) were less than those reported in the Impulse Control Disorders and the Association of Neuropsychiatric Symptoms, Cognition and Quality of Life in Parkinson Disease (ICARUS) study^28^ (a similarly large observational study), which reported a prevalence of 32% to 35% of the cohort screening positive for PD-ICB, which remained stable across 2 years. In contrast, a recently published longitudinal study^29^ (with a younger cohort [mean age 62 years]) found a prevalence rate of 19.7% at baseline, which increased to 32.8% after 5 years. Factors associated with increased prevalence in that cohort included disease duration and dose of DA used. These differences may be explained by the younger sample and much higher proportion on DAs at baseline compared to this cohort (74% vs 30%), thus having a higher likelihood of developing ICB.

The in-depth PICS interview allows greater accuracy in delineating subdomains compared to the QUIP screening questionnaire. Pathologic gambling was present in only 1 participant in the in-depth interview, which may reflect a particular difficulty in engaging with this group or greater responsivity to medication alteration. The authors noted that during the in-depth interview many of the patients with hobbyism reported technology overuse as a major symptom, including smartphone games and searching the internet. These device applications are designed to encourage repeated use, so this may be particularly problematic for those vulnerable to ICBs.^30^

### Severity of ICBs

Relationship difficulties, including marital separation, are common in moderate to severe PD-ICD and can be associated with subsyndromal behaviors. The overlap of moderate symptom severity between both the subsyndromal ICD and the full ICD group highlights the importance of recognizing the full spectrum of these disorders and how the impact of the behaviors can have a lasting detrimental effect on an individual's quality of life despite apparent improvement in the symptoms. It also highlights the need for research in this larger group of patients with PD-ICB.

In the follow-up interview, there was significant variation in both the impact and intensity of PD-ICB, and thus the severity, within a relatively short time frame. Many even changed category between ICD, subsyndromal ICD, and asymptomatic. This variation was not due to changes in DA use or medication dose and contrasts with large prevalence studies that found a positive relationship between PD-ICD and total LEDD.^4,31^ Further studies designed to examine this relationship have found an association with total DA dose.^29,32^ However, these studies used categorical outcomes that may not necessarily have reflected the dimensional approach of the semistructured interview.

In addition, the in-depth interviews allowed preliminary exploration of possible subtypes of PD-ICB. Many of the patients with subsyndromal PD-ICD described that their symptoms were preexisting behaviors (before medication initiation) that had worsened, e.g., worsening of symptoms in those with a tendency to heavier internet use or a proclivity to comfort eating after titration of their PD medication. In contrast, cases with syndromal PD-ICD often described a relatively rapid onset of a behavioral change that the participants described as new or out of character (e.g., a change in sexual orientation or a novel gambling habit) after the initiation of a DA. The first subtype may experience a gradual worsening of preexisting ICB symptoms in a dose-responsive fashion to dopaminergic medication, while a different group may have a predisposition to a more rapid and catastrophic PD-ICD, leading to conflicting results.

Some participants met with their physician, who felt that withdrawal in the context of only mild symptoms, on balance, was not necessary. The common difficulties of agonist withdrawal (e.g., DA withdrawal syndrome^33^) were described by some patients during the follow-up interview. Some managed only a modest reduction in DA or a switch to a weaker agonist preparation. This highlights the need for other forms of intervention in PD-ICB such as cognitive behavioral therapy to allow a more holistic approach.^34^

Medication was not the only factor to influence the severity of PD-ICB. The qualitative data recorded suggested an important role for internal factors (mood and coping mechanisms) and external factors (major life events and social support networks), consistent with the literature.^35^ Addressing these factors may help manage the full spectrum of PD-ICB, preventing the transition from subsyndromal to ICD, and may even allow more modest reductions in medications and thus better symptom control. PD-ICD increases caregiver burden over PD alone,^36^ and better social functioning increases the chances of improvement in severity.^37^

The incidence of depression is higher among participants with PD with ICD than those without.^28,38^ A study^39^ that used objective quantitative measures of psychological factors such as mood, anxiety, and a lack of healthy coping strategies found that these factors were significant predictors of PD-ICD incidence. The causal relationship between ICB and such factors is difficult to determine, but a conceptual model^40^ proposes that the effect of underlying psychosocial stressors is multiplied by the effect of DAs to cause PD-ICD. This is supported by evidence that psychotherapeutic strategies such as cognitive behavioral therapy^35^ and support groups^41^ are effective in reducing the severity of both mood disorders (such as depression and anxiety) and ICD itself.

### Factors associated with ICBs

While the precise etiology of PD-ICB remains unclear, the ability to predict individuals who are at risk for developing these behaviors, and thus avoiding precipitating medication, would have immediate clinical utility. Consistent with previous studies, the use of dopaminergic medication, particularly DAs, was most strongly associated with the presence of ICBs. The severity of motor complications was also independently associated with ICBs. These motor associations may at least in part be explained by motor progression of advancing PD or an increasing LEDD, although these were not independently associated with PD-ICB. Motor symptoms, as measured by the MDS-UPDRS III, have been inconsistently associated with PD-ICB, but this has been largely in studies using screening tools for diagnosis as opposed to a formal interview. The ALTHEA study^42^ similarly found a strong association between dyskinesia and PD-ICB, although the reported rates were higher in that study due to the use of a screening tool for diagnosis.

Similar to the ICARUS study, apathy was associated with PD-ICB.^28^ The replication of apathy being associated with the presence of PD-ICB is intriguing in that it is linked to reward insensitivity, which is modulated by dopaminergic medication.^43^ Apathy is increasingly recognized in PD, and it may be that disruption of these same pathways between the prefrontal cortex and basal ganglia, which are integral to reward sensitivity, causes a susceptibility to ICD. Mood disorders such as anxiety and depression, which have been associated with PD-ICB, correlate strongly with apathy, so previous studies may have associated these potential confounding variables. The alternative, given that these patients seem to have a high drive and motivation for reward-based activities, is that this observation may in fact represent a “pseudo-apathy” in which, akin to a substance user, patients lose interest in non–substance use–related reward activities.

In contrast to previous studies, we did not identify age, sex, cognition, sleep disorders, and marital status as risk factors in the PD group. PD-RBD was identified as an independent risk factor in a large cross-sectional study.^8^ This finding was then replicated in cohorts with both clinically^44^ and polysomnography^7^-diagnosed PD-RBD. Further studies with smaller sample sizes,^45–47^ aiming to specifically address this question, have failed to show this association. This large study adds robust evidence for the lack of association between PD-RBD and PD-ICD.

### ICBs in RBD

Despite many participants with RBD and controls screening positive on the QUIP, only 1% of the total of each group met the criteria for ICD, indicating that RBD in itself is not a risk factor. The increased reporting of potentially abnormal behaviors on the self-completed screening questionnaire (QUIP) by the RBD group compared to controls may reflect a reporting bias. Patients with RBD are aware that they are at risk of developing a neurodegenerative disease such as PD and may therefore overestimate their symptoms. Although the mechanisms for ICD are not yet fully understood, the primary driver is thought be dopaminergic medication, to which the RBD group was not exposed. In addition, studies investigating the pathophysiology of ICD implicate abnormal signaling of dopaminergic projections into the striatum, orbitofrontal cortex, anterior cingulate, and anterior insula, areas that are not implicated in the pathology of RBD.^48,49^

Major strengths of our study include the following: one of the largest prospective studies to examine PD-ICB; the adoption of a dimensional compared with the more categorical approach to this range of disorder; the inclusion of patients with incident cognitive impairment, because their exclusion may bias the findings to less aggressive PD subtypes (thus, this unselected cohort is highly representative of early PD); and the use of recognized diagnostic criteria based on an in-depth interview that allows greater accuracy of diagnosis compared to screening questionnaires. Study limitations include the ≈50% participation rates for the in-depth interview, although the literature indicates that patients with PD-ICB are in fact significantly more likely to respond to a follow-up survey than patients with PD without ICBs.^6^ The additional use of multiple imputation mitigates against potential bias in response rate, in contrast to the many ICD prevalence studies that merely reported the crude observed prevalence. A second limitation is that there may still be residual confounding differences in determining risk factors even though we have used a multivariable model. Finally, the relatively small absolute number of patients with ICBs for follow-up may prove a limitation. However, the in-depth interview allows a significant amount of detail to be collated on each participant.

This study highlights the importance and potential impact across the full spectrum of severity of PD-ICB symptoms. Long-term management of these symptoms is challenging because symptoms persist and may even worsen, highlighting the need for holistic care, including nonpharmacologic therapies.

## Glossary

CI: confidence interval
DA: dopamine agonist
DSM-5: *Diagnostic and Statistical Manual of Mental Disorders, 5th edition*
ICARUS: Impulse Control Disorders and the Association of Neuropsychiatric Symptoms, Cognition and Quality of Life in Parkinson Disease
ICB: impulse control behavior
ICD: impulse control disorder
LEDD: levodopa equivalent daily dose
MDS-UPDRS: Movement Disorder Society–revised Unified Parkinson's Disease Rating Scale
PICS: Parkinson's Impulse-Control Scale
QUIP: Questionnaire for Impulse Control Disorders in Parkinson's Disease
QUIP-S: QUIP Short Form
RBD: REM sleep behavior disorder
